# Minimising radiation exposure in catheter ablation of ventricular arrhythmias

**DOI:** 10.1186/s12872-021-02120-4

**Published:** 2021-06-16

**Authors:** Matevž Jan, David Žižek, Tine Prolič Kalinšek, Dimitrij Kuhelj, Primož Trunk, Tadeja Kolar, Juš Kšela, Martin Rauber, Mehmet Yazici

**Affiliations:** 1 Cardiovascular Surgery Department, University Medical Centre Ljubljana, Zaloška 7, 1000 Ljubljana, Slovenia; 2 Cardiology Department, University Medical Centre Ljubljana, Ljubljana, Slovenia; 3 Clinical Institute for Radiology, University Medical Centre Ljubljana, Ljubljana, Slovenia

**Keywords:** Ventricular arrhythmia, Radiation exposure, Catheter ablation, Zero-fluoroscopy

## Abstract

**Background:**

Conventional fluoroscopy guided catheter ablation (CA) is an established treatment option for ventricular arrhythmias (VAs). However, with the complex nature of most procedures, patients and staff bare an increased radiation exposure. Near-zero or zero-fluoroscopy CA is an alternative method which could substantially reduce or even eliminate the radiation dose. Our aim was to analyse procedural outcomes with fluoroscopy minimising approach for treatment of VAs in patients with structurally normal hearts (SNH) and structural heart disease (SHD).

**Methods:**

Fifty-two (age 53.4 ± 17.8 years, 38 male, 14 female) consecutive patients who underwent CA of VAs in our institution between May 2018 and December 2019 were included. Procedures were performed primarily with the aid of the three-dimensional electro-anatomical mapping system and intra-cardiac echocardiography. Fluoroscopy was considered only in left ventricular (LV) summit mapping for coronary angiography and when epicardial approach was planned. Acute and long-term procedural outcomes were analysed.

**Results:**

Sixty CA procedures were performed. Twenty-five patients had SHD-related VAs (Group 1) and 27 patients had SNH (Group 2). While Group 1 had significantly higher total procedural time (256.9 ± 71.7 vs 123.6 ± 42.2 min; *p* < 0.001) compared to Group 2, overall procedural success rate [77.4% (24/31) vs 89.7% (26/29); *p* = 0.20)] and recurrence rate after the first procedure [8/25, (32%) vs 8/27, (29.6%); *p* = 0.85] were similar in both groups. Fluoroscopy was used in 3 procedures in Group 1 where epicardial approach was needed and in 4 procedures in Group 2 where LV summit VAs were ablated. Overall procedure-related major complication rate was 5%.

**Conclusions:**

Fluoroscopy minimising approach for CA of VAs is feasible and safe in patients with SHD and SNH. Fluoroscopy could not be completely abolished in VAs with epicardial and LV summit substrate location.

## Background

Catheter ablation (CA) guided by conventional fluoroscopy (CF) is an established treatment option for ventricular arrhythmias (VAs) [1]. However, the use of fluoroscopy exposes patients and medical staff to potentially harmful stochastic and deterministic effects of ionising radiation [2–4]. This risk is significantly increased in long-lasting, complex, and multiple ablation procedures like CA of atrial fibrillation (AF) and VAs, with 60 min of fluoroscopy resulting in a lifetime risk of 0.07% for women and 0.1% for men for fatal malignancy [5–7]. Reduction of radiation exposure during CA procedures with employing the ALARA (as low as reasonably achievable) principles is an already established routine in electrophysiology laboratories [8].

In recent years, reports of near-zero (NZF) and zero-fluoroscopy (ZF) procedures show that it is possible to significantly reduce or even eliminate radiation exposure in most CA procedures. Fluoroscopy minimising approaches utilising three-dimensional (3D) electro-anatomical mapping (EAM) systems have been frequently used in children and adults with supra-ventricular tachycardias (SVTs) and in adults with AF [9–18]. However, reports of NZF or ZF approaches in CA of different forms of VAs are relatively scarce [19–21].

Our aim was to analyse procedural outcomes with fluoroscopy minimising approach for treatment of VAs in patients with structurally normal hearts (SNH) and structural heart disease (SHD).

## Methods

### Study population

Consecutive patients who underwent CA of VAs in our institution between May 2018 and December 2019 were included. In general, patients with SNH and idiopathic VAs, as well as patients with ventricular tachycardias (VT) resulting from SHD were included.

Indications to undergo CA were followed according to the published guidelines and consensus documents [1]. Included patients with SNH had an ECG documentation of ventricular ectopy, with an estimated daily ectopy burden of at least 10%, or tachycardia that were causing either severe symptoms unresponsive to anti-arrhythmic drugs (AADs), or presumed tachycardia-induced cardiomyopathy. All included patients with SHD had a documented sustained VA either with 12-lead ECG or a cardiac implantable electronic device (CIED).

### Pre-procedural management

Trans-thoracic echocardiogram was performed in all patients before the CA procedure. Pre-procedural computed tomography (CT) or magnetic resonance imaging (MRI) were accessible in most patients, however, images obtained were not used to create three-dimensional reconstructions for procedural purposes. All patients with a suspected SHD had pre-procedural assessment of coronary anatomy with invasive coronary angiography or CT angiography. Combined results of imaging studies were used to assess the presence of SHD.. Basic laboratory blood tests, clinical examination and CIED interrogations were also performed in all patients.

### Procedural setup

Procedures were preferably performed in conscious sedation, except for patients undergoing epicardial approach in whom general anaesthesia was used. Femoral vein and artery punctures were performed to access the heart. A 3D EAM system (Carto®, Biosense Webster, Irvine, California, USA or NavX™ Precision™, Abbott, Abbott Park, Illinois, USA) and intra-cardiac echocardiography (ICE) (Acuson AcuNav™, Biosense Webster, Irvine, California, USA) were routinely used simultaneously. In patients with left-sided arrhythmias, we generally used the ante-grade trans-septal approach, except when mapping and ablation in the aortic root were needed in which case a retrograde trans-aortic approach was used. A steerable long sheath (Agilis™, LRG Curl, Abbott, Abbott Park, Illinois, USA) was used to improve catheter stability in trans-septal approach. Typically, uni-directional irrigated tip radio-frequency (RF) ablation catheters with (Thermocool Smarttouch® and Thermocool Smarttouch® SF, both Biosense Webster, Irvine, California, USA) and without (FlexAbility™, Abbott, Abbott Park, Illinois, USA) the ability of contact force sensing were utilised. In patients with SHD and suspected substantial arrhythmogenic substrate we used a multipolar catheter enabling high-density mapping (PentaRay®, Biosense Webster, Irvine, California, USA).

### Procedural workflow

Once all catheters and the ICE probe were inserted, we followed two basic protocols depending on the type of arrhythmia.

1. In patients with a SHD and a recorded VA, the first step was VT induction with programmed ventricular stimulation. The next step was substrate mapping with the ablation catheter and later detailed mapping with the multipolar mapping catheter. Anatomical and voltage maps of the chamber of interest were created with the 3D EAM system and all electrograms with characteristics of local abnormal ventricular activity (LAVA) [22] and obvious late and diastolic electrical potentials (LP) were tagged [23]. Additionally, the areas of the substrate with suspected LAVA and without obvious LP were mapped during decremental RV pacing to reveal hidden slow conduction, which was also tagged [24]. Substrate mapping was combined with limited pace-mapping to reveal the exit point of the induced VT, with adjacent low voltage areas thoroughly mapped. Finally, all areas with late potentials and hidden slow conduction were extensively ablated using the described irrigated catheters with power-controlled settings of 30–45 W for at least 40 s or until the disappearance or at least attenuation of the local electrocardiogram. A re-mapping of the ablated parts of the substrate and surrounding areas with the multipolar mapping catheter was routinely performed and additional ablations were done if needed to achieve complete substrate modification, i.e. absence of local electrical activity. Endpoints of the procedure were substrate modification and non-inducibility of VT with programmed ventricular stimulation with up to four delivered extrasystoles combined with isoproterenol infusion.
2. In patients with presumably idiopathic VAs only very partial anatomical and voltage maps were created with the 3D EAM. Typically, our strategy relied on activation mapping of the present ventricular ectopy or VT. Pace-mapping was also used to complement activation mapping when needed. The mapped origin of the arrhythmia was ablated with similar settings and parameters as described in patients with SHD. The endpoint was termination of the ectopy or tachycardia. An additional endpoint was the absence and non-inducibility of VAs with fast and programmed ventricular stimulation, also after infusion of isoproterenol.

### Epicardial approach

When epicardial mapping was needed a steerable long sheath (Agilis EPI™, Abbott, Abbott Park, Illinois, USA) was inserted over the J-tip guide-wire through surgically prepared sub-xiphoid epicardial approach.

### Fluoroscopy minimising approach

Procedures were generally performed without any fluoroscopy and only with the use of the 3D EAM system and ICE. Fluoroscopy was only considered in two sets of cases: 1. In LV summit mapping for coronary angiography to avoid RF delivery in close vicinity of a coronary arteries. 2. In procedures where the epicardial approach for mapping and ablation was planned. Fluoroscopy time and dose area product (DAP) were measured and reported for each procedure.

### The use of intra-cardiac echocardiography

ICE was used in conjunction with the 3D EAM system in all procedures. ICE had four basic roles:

1. Providing guidance of guide-wire, long sheath, and trans-septal needle during trans-septal puncture (TSP) as previously described [25].
2. Providing additional imaging information about heart anatomy relevant for accurate and effective endocardial mapping and ablation. Examples include: achieving catheter stability at certain regions of the ventricles such as papillary muscles and moderator band; observing direction and position of the long sheath and catheter loops in cases of mapping and ablation at the base of the ventricles.
3. Replacing coronary angiography for visualisation of coronary artery ostia during ablation in the region of the sinuses of Valsalva.
4. Timely detection of possible complications such as sudden tissue whitening preceding “steam pops”, pericardial effusion, potential thrombotic masses in the heart cavities or on sheaths and catheters inserted into the heart, sudden deterioration of systolic function of the ventricles.

### Definition of procedural parameters, procedural success, complications and follow-up

The procedural duration (total procedural time, TPT) was defined as the time interval from the placement of the venous and arterial sheaths to their removal at the end of the ablation procedure. In the case of a surgical sub-xiphoid epicardial approach time measurement was started with skin incision. Ablation time [time spent delivering radio-frequency (RF) energy to the tissue)] and the number of ablations were automatically recorded by the intra-cardiac electrogram registration and analysis system. Procedures were considered successful if predefined endpoints were reached. Overall procedural success rate (OSR) was defined as procedural success of combined first and repeat procedures for treated VA. Recurrence rate (RR) was defined as recurrence of the treated VA after the first procedure. Any early (during the same hospital stay) and late complications were reported as complication rate (CR), calculated as percentage of procedures. Major complications were all adverse events resulting in procedure termination, prolonged hospital stay, long-term disability, and any additional intervention and bleeding requiring transfusion.

Patients were followed clinically and with the 12-lead ECG recordings 3 months after the procedure and every 6 months thereafter. Patients with a CIED had its telemetry examined at every visit for possible episodes of VT, appropriate and inappropriate interventions, such as anti-tachycardia pacing (ATP), direct current cardioversions, and defibrillations. In patients treated for sustained VT, it's recording during the follow-up (ECG or CIED tracing) was regarded as a recurrence. In patients treated for ventricular ectopy, 24-h holter recordings were typically used to assess the daily VAs burden at least one month after the ablation procedure. If the VAs burden was comparable to the one expected in the normal population, patients were considered to be cured and without recurrence. Procedures were considered successful if expected endpoints were reached. Any recurrences were used to calculate the recurrence rate (RR).

### Statistical analysis

The statistical analyses were carried out using the IBM SPSS software version 24.0 for Windows (IBM Corporation, Armonk, NY, USA). Continuous variables were presented as mean ± standard deviation (SD) while categorical variables were presented as number and percentage. Differences between groups were analysed using unpaired Student’s t-test for numeric variables and Pearson Chi-square test and Fisher exact test for attributive variables. Multiple group comparisons were performed with one-way ANOVA. *p* value was considered significant when it was less than 0.05.

## Results

Fifty-two (age 53.4 ± 17.8 years, 38 males, 14 females) patients had 60 CA procedures for treatment of VAs. Twenty-five patients had SHD-related VAs (Group 1), among which 19 patients had VT due to ischaemic cardiomyopathy (ICM), 6 had ventricular arrhythmia/tachycardia due to non-ischaemic cardiomyopathy (NICM) and 1 had a combination of congenital heart disease and ischaemic heart disease (IHD). There were 27 patients with SNH and idiopathic VAs (Group 2). Detailed baseline demographic and clinical characteristics are presented in Table 1.

**Table 1:** Demographic and clinical characteristics of the study population.

	All (n = 52)	Group 1 (n = 25)	Group 2 (n = 27)
Age (years)	53.4 ± 17.8	62 .8 ± 12.5	44.7 ± 17.7*
Male gender [number (%)]	38 (73.1)	24 (96%)	14 (51.9)*
Body Mass Index (kg/m^2^)	25.6 ± 5.2	27.1 ± 3.8	24.2 ± 6.0**
Arterial hypertension [number (%)]	27 (51.9)	19 (76)	8 (29.6)
Hyperlipidemia [number (%)]	27 (51.9)	19 (76)	8 (29.6)
Diabetes mellitus [number (%)]	5 (9.6)	3 (12)	2 (7.4)
Ischaemic cardiomyopathy [number (%)]	19 (36.5)	19 (76)	0
Non-ischaemic cardiomyopathy [number (%)]	5 (9.6)	5 (20)	0
Congenital heart disease [number (%)]	1 (1.9)	1 (4)	0
LVEF (%)	49.5 ± 13.1	40.8 ± 10.4	57.5 ± 9.7*
LVEDV (ml)	154.5 ± 62.4	194.7 ± 64.3	117.1 ± 28.5*
RV dysfunction [number (%)]	5 (9.6)	4 (16)	1 (3.7)
CIED present[number (%)]	16 (30.7)	16 (64)	0
AAD non-amiodarone [number (%)]	34 (65.3)	21 (84)	13 (48.1)
Amiodarone [number (%)]	9 (17.3)	7 (28)	2 (7.4)
Sustained VT [number (%)]	15 (28.8)	10 (40)	5 (18.5)
Electrical storm [number (%)]	8 (15.4)	8 (32)	0
Tachycardia induced cardiomyopathy [number (%)]	4 (7.6)	0	4 (14.8)

*p < 0.001; group I versus Group II, **p < 0.05; group I versus Group II

*LVEF* left ventricular ejection fraction, *LVEDV* left ventricular end-diastolic volume, *RV* right ventricular, *CEID* cardiac implantable electronic device, *AAD* anti-arrhythmic drugs, *VT* ventricular tachycardia

### Procedural parameters

Group 1 had significantly higher TPT compared to group 2 (256.9 ± 71.7 and 123.6 ± 42.2 min; *p* < 0.001) (Table 2). Overall procedural success rate (OSR), including the repeat procedures, was 83% (50/60) for the entire study population. OSR was similar in both groups [77.4% (24/31) and 89.7% (26/29); *p* = 0.20)].

**Table 2:** Procedural characteristics

	Group 1 (n = 31)	Group 2 (n = 29)
Septal substrate related VT [number (%)]	8 (25.8)	–
Epicardial substrate related VT [number (%)]	6 (19.4)	–
Outflow tract ectopy/VT [number (%)]	–	18 (62.1)
Fascicular VT [number (%)]	–	2 (6.9)
Moderator band ectopy/VT [number (%)]	–	2 (6.9)
Postero-septal process ectopy/VT [number (%)]	–	4 (13.8)
Papillary muscle ectopy/VT [number (%)]	–	1 (3.4)
Other substrate/origin locations [number (%)]	28 (90.3)	4 (13.8)
Total procedural time (min)	256.9 ± 71.7	123.6 ± 42.2*
Number of ablations (number)	80.9 ± 40.5	15.4 ± 8.6*
Total ablation time (seconds)	3240 ± 1690	537 ± 357 *
Trans-septal punctures [number (%)]	28 (90.3)	8 (27.6) *
Epicardial mapping and ablation [number (%)]	3 (9.7)	4 (13.8)
Procedures with high density mapping [number (%)]	25 (80.6)	4 (13.8) *
Procedures with general anaesthesia [number (%)]	6 (19.4)	4 (13.8)
Overall Procedural Success Rate [number (%)]	24 (77.4)	26 (89.7)
Repeat procedures [number (%)]	6 (19.4)	2 (6.9)
Procedures with fluoroscopy [number (%)]	3 (9.7)	4 (13.8)
Fluoroscopy time (min)	21.2 ± 5	8.3 ± 4
DAP per procedure [Gycm^2^ (SD)], if fluoroscopy used	48.6 ± 23 (3 procedures)	25.4 ± 14 (4 procedures)

*VT* ventricular tachycardia, *DAP* dose area product, *SD* standard deviation

*p < 0.001; group I versus Group II

### Procedural outcomes

During the follow-up period of 255 ± 170 days, there were 16 recurrences after the first CA procedure (16/52, RR 30.7%). RR after the first procedure was similar in both groups [8/25 (32%) and 8/27 (29.6%); *p* = 0.85]. Detailed information on procedural findings and parameters are presented in Table 2.

All idiopathic VAs originating from the RV (12 from RVOT) were successfully ablated (16/16, 100%) and there were no arrhythmia recurrences during follow-up. In 9 patients with VAs originating from LV 77.7% (7/9) procedural success rate was recorded and there were 2 recurrences (22.2%) after the initial ablation. In patients with NICM and ICM, OSR [respectively; 62.5% (5/8) and 82.6% (19/23); *p* = 0.18)] and RR after the first ablation [respectively; 33.3% (2/6) and 26.3% (5/19); *p* = 0.27)] were similar.

Patients with failed procedures in Group 1 included one patient with IHD (intramural substrate) that had 3 procedures including epicardial mapping and ablation and one patient with NICM (post-myocarditis aetiology of intramural ectopy substrate) that had two failed endocardial procedures. In both patients arrhythmia occurrence was successfully controlled with amiodarone. There were two ICM patients (epicardial origin of arrhythmia) with one failed endocardial procedure. After bisoprolol up-titration they did not require repeat procedures. Similarly, patients with failed procedures in Group 2 included one patient with LV-summit ectopy that was successfully ablated after repeat procedure; one patient with ectopy from LV postero-septal process that was successfully ablated at the repeat procedure; one patient with ectopy from the commissure between the left and right aortic cusps that refused repeat ablation and whose ectopy was apparently sufficiently suppressed to enable normalisation of previously reduced systolic function of LV during follow-up. One patient with ICM that had CA due to a VT storm shortly after emergency heart surgery died approximately one month after the ablation procedure because of heart surgery related complications.

### Use of fluoroscopy

**Fig. 1:**
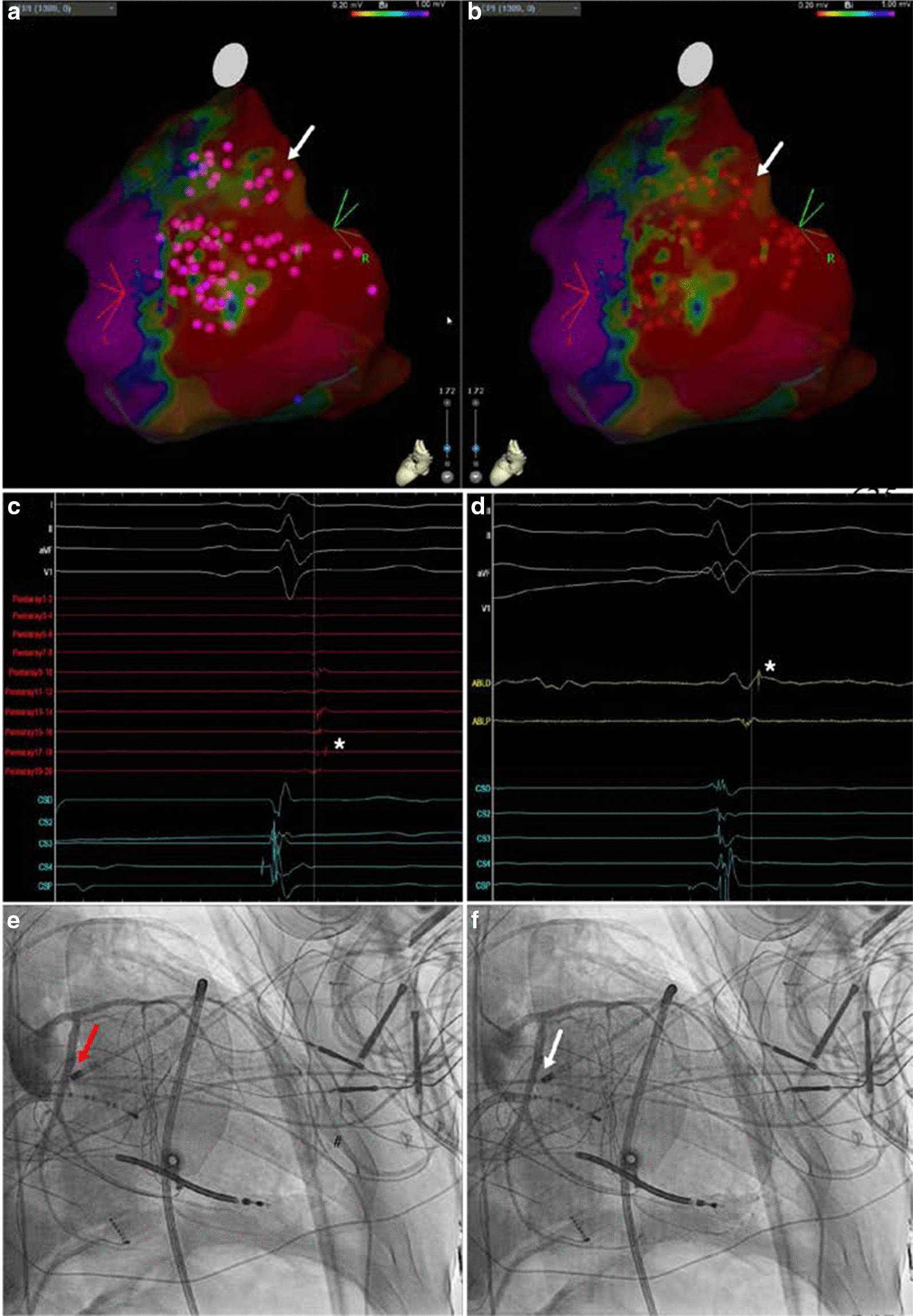
Epicardial ablation. (**A**, **B**) Both present a modified left lateral view of a partial three-dimensional (3D) electro-anatomical mapping (EAM) voltage map of the epicardial part of the left ventricle (LV) with purple dots representing local abnormal ventricular activity (LAVA) sites inside and on the border of the low voltage area and red dots representing eventual ablation lesions. The white arrows mark the approximate location of the recorded LAVA as shown on intra-cardiac electrogram recordings made with multipolar mapping catheter presented on (**C**) (Pentaray 1–20) and with ablation catheter presented on (**D**) (ABLD and ABLP). The white asterisks mark the recorded isolated diastolic potentials at one of the eventual ablation sites near the course of the left circumflex artery (LCX) at the base of the epicardial side of the left ventricle. Before ablation at that site, selective left coronary angiography in a modified right anterior oblique view was performed as shown on (**E**, **F**). Red arrow on (**E**) points at the tip of the ablation catheter touching the proximal part of the LCX in which case the catheter was slightly withdrawn to the site marked with the white arrow on (**F**) showing the tip at the safe distance from the LCX at the location of the eventual successful ablation. CSD-CSP intra-cardiac electrograms recorded with a 10-polar diagnostic catheter in the right ventricle; I, II, aVF and V1 surface electrograms

**Fig. 2:**
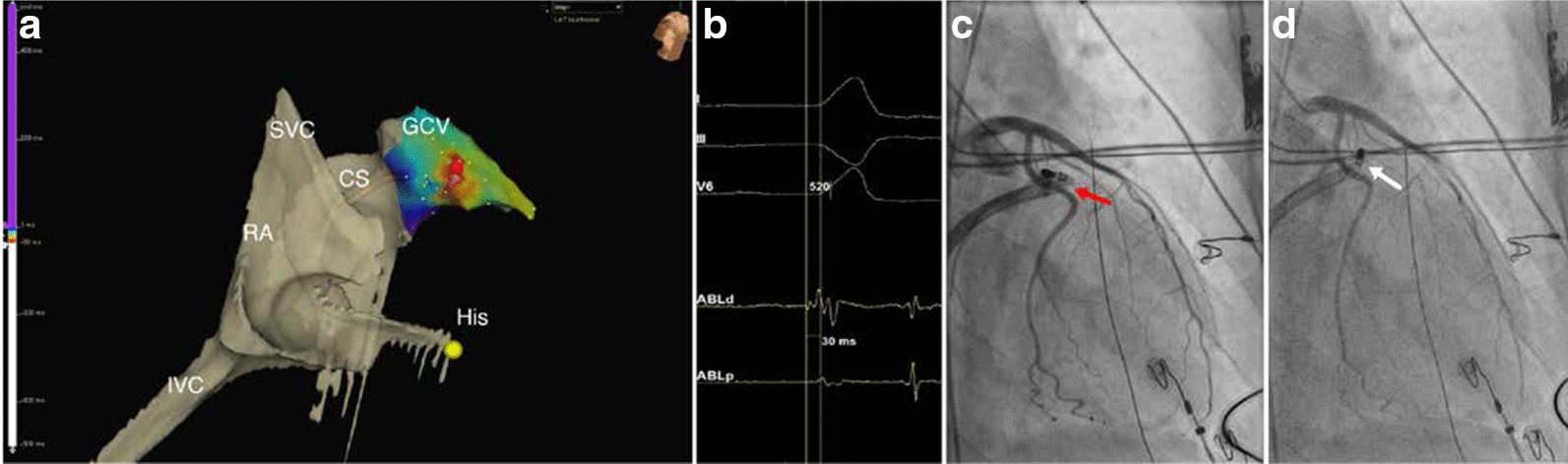
Left ventricular summit area ablation. (**A**) A modified right anterior oblique (RAO) view of a partial three-dimensional (3D) electro-anatomical mapping (EAM) activation map of the right atrium (RA), the coronary sinus (CS) and its continuation into the great cardiac vein (GCV) where the earliest local ventricular activation during left ventricular (LV) summit ventricular ectopy was found and successfully ablated (red dot). (**B**) Surface (I, III and V6) and intra-cardiac electrograms (ABLd and ABLp) revealing the earliest local ventricular activation (− 30 ms) during LV summit ectopy as recorded in the GCV. Before actual ablation at the earliest activation site selective left coronary angiography in a modified RAO view was performed as shown on (**C**, **D**). Red arrow on (**C**) points at the tip of the ablation catheter touching the proximal part of the LCX in which case the catheter was slightly withdrawn to the site marked with the white arrow on (**D**) showing the tip at the safe distance from the LCX at the location of the eventual successful ablation. His marks the His bundle location; SVC superior vena cava; IVC inferior vena cava

Three procedures in Group 1 (3/31, 10%) required additional X-ray fluoroscopy that was used for 21.2 ± 5 min with DAP of 48.6 ± 32 Gycm2. In all three procedures endocardial and epicardial ablation were performed (Fig. 1). Two procedures were repeat procedures in patients with ICM. One procedure was a first-time CA in a patient with arrhythmogenic cardiomyopathy of RV and LV. Procedural success was achieved in two (2/3, 66%) epicardial procedures. There were no arrhythmia recurrences, even in the procedure that was considered unsuccessful, however, this patient was eventually treated with amiodarone. Four procedures (4/29, 14%) in Group 2 required X-ray fluoroscopy that was used for 8.3 ± 4 min with DAP of 25.4 ± 14 Gycm2. In all four procedures LV-summit area was the origin of VA and epicardial mapping and ablation was needed in the great cardiac vein (Fig. 2). Additionally, coronary angiography had to be performed to avoid RF delivery close to coronary artery. All four (4/4, 100%) procedures were acutely successful with one recurrence during follow-up. Four VA ablation procedures in Group 2 were successfully performed in paediatric patients without the use of X-ray fluoroscopy.

### Procedural adverse events

As shown in Table 3 there were overall 3 (3/60, 5%) major complications related to the procedures. One patient with an ICM had perforation of RV during an attempted surgical approach to the epicardium. The perforation was also treated surgically. One patient had a late pericardial tamponade occurring 1 week after successful ablation of idiopathic RVOT ectopy which required percutaneous drainage. One patient with an ICM had pseudo-aneurysm of the left femoral artery that was treated surgically.

**Table 3:** Procedural complications

	All (n = 60)	Group 1 (n = 31)	Group 2 (n = 29)
Stroke/TIA	0	0	0
Pericardial tamponade	1 (1.7)	0	1 (3.4)
RV perforation	1 (1.7)	1 (3.2)	0
Pneumothorax	0	0	0
Hematothorax	0	0	0
PSA surgery/intervention	1 (1.7)	1 (3.2)	0
CIED lead dislocation	0	0	0
Overall	3 (5)	2 (6.4)	1 (3.4)

*TIA* transient ischaemic attack, *RV* right ventricular, *PSA* arterial pseudo-aneurysm, *CIED* cardiac implantable electronic device

## Discussion

The main findings of our study show that fluoroscopy minimising approach for CA procedures can be safely and efficiently performed in most patients with VAs. Procedural outcomes of this approach were similar in patients with SHD and SNH. Fluoroscopy was only used in 3 SHD patients with epicardial substrate and in 4 SNH cases with LV summit substrate where intra-procedural coronary angiography was needed to avoid RF delivery in close vicinity of coronary arteries.

The main findings of our study are consistent with the results of earlier studies on efficacy and safety of CF-based CA in patients with and without SHD [26–28]. In a prospective study involving 227 adults with SHD (63 NICM and 164 ICM) and VT, Dinov et al. [26] demonstrated that the non-inducibility of VT was achieved in 66.7% of NICM and in 77.4% of ICM patients that were ablated with the aid of CF combined with the 3D EAM system. Complication rate in both groups was similar (11.1% for both groups), however, higher compared to ours (8.6% for ICM and 0% for NICM). In addition, reported VT-free survival rates (40.5% in NICM and 57% in ICM) at 1-year follow-up were lower compared to our results (66.7% and 73.7%; respectively). Although different study population might have impacted on the discrepancy of the results, it is reasonable to assume that ZF approach does not compromise procedural efficacy. Furthermore, Tanawuttivat et al. reported that CF guided CA acute success rate varied from 76 to 100%, RR was 0–23% and CR was 0–3% in 815 patients with idiopathic VAs originating from outflow tracts [27]. In line with these observations, our CA success rate in VAs originating from RVOT was 100%, with 0% RR and 3.4% CR. In addition, ZF approach in our study yielded similar OSR (89.7% in SNH, 82.6% in ICM, and 62.5% in NICM patients; *p* < 0.001) compared to the CF guided CA of VAs reported by Kumar et al. [28] (OSR 79%, 56%, and 60%, respectively; *p* < 0.001) where patients with and without SHD were compared. These observations further affirm the feasibility of ZF approach in various patient groups with VAs.

It has to be recognised that data on completely ZF approaches for CA for VAs is relatively scarce. In some studies, involving a small number of patients with VAs (≤ 20), it has been shown that all CA procedures have been successfully completed with ZF approach and no complications [12, 13]. In their single-centre experience with a completely ZF approach using the 3D EAM system and ICE in patients with idiopathic VAs, Lamberti et al. [29] and Sanchez et al. [12] determined similar findings regarding outcome and safety. All procedures could be completed without fluoroscopy and reasonable TPT values (respectively; 150 ± 45 min and 170.2 ± 45.7 min). Additionally, Razminia et al. [13] reported successful completion of ZF CA in 10 patients with ICM related VTs. Similar to our results, they also remarked that median TPT was 257.5 min and RR was 21.4%. Moreover, authors of some large studies including patients with different types of arrhythmias, predominantly SVTs, and very few patients with VAs, reported that the use of the ZF approach was possible in 20–100% of procedures, with an ASR of 80–100%, with a TPT of 128 ± 21 min–169 ± 43.2 min, and CR of 0–2.5% [11, 14, 30, 31]. These studies based on single experienced operators or single-centre reports do not have enough statistical power to enable analysis of factors related to the efficacy and safety of ZF CA. Also, the discrepancies between all study results, including ours, can be explained, at least in part, by differences in study design, patient population, VA substrate location, 3D EAM system utilisation, and in follow-up duration.

In a recent large-scale study comparing CF and ZF approach in patients with idiopathic VAs [19] it was implied that all idiopathic VAs cannot be successfully and safely treated completely without the use of fluoroscopy. In this prospective multi-centre study Wang et al. enrolled 489 patients with idiopathic VAs and reported that there was no significant difference between the ZF approach (n = 163) and CF approach (n = 326), including ASR (84.1%vs 85.4%, respectively), RR (1.9% vs 2.2%), or severe CR (0.6% vs 0.9%). In line with our observations, there were 9 cases where fluoroscopy was used for coronary angiography. Although it is the largest study on the subject, it is not randomised and, more importantly, excluded patients with SHD. Additionally, follow-up to determine the arrhythmic recurrence was shorter (5.4 ± 3.9 months) compared to our study. In a study that enrolled patients with SHD (22 ICM and 19 NICM patients), Cano et al. suggested that a significant (but not complete) reduction in radiation exposure can be achieved by using the CARTOUNIVU module in scar-related VT ablation procedures [20]. Median fluoroscopy time in the study population was 6.1 min and DAP 10.8 Gycm^2^, with higher reported radiation exposures in NICM patients and in epicardial procedures. Compared to our study, completely ZF procedures were not performed and ASR in patients with SHD was somewhat lower (66% vs.77%, respectively). However, it has to be recognised that the advantage of the CARTOUNIVU module seems to be in integration of coronary angiography images with the 3D EAM system, thus lowering the fluoroscopy time and DAP in cases where use of X-ray cannot be completely avoided.

The characteristics of the main studies used for discussion purposes are presented along with our findings in the Table 4.

**Table 4:** Main characteristics of studies used for discussion purposes

Author, year	ZF/MinF/F	Number of patients	Type of study	Type of VA	Procedural duration	OSR	RR	CR	Epicardial ablation, % of procedures	Use of fluoroscopy	Fluoroscopy time and dose	Use of ICE, % of procedures
Our study, 2021	MinF	52	Retrospective, consecutive pts	PVC, Idiopathic VT and SHD related VT (NICM, ICM)	123.6 ± 42.2 min for PVC and idiopathic VT, 256.9 ± 71.7 min for SHD related VT	89.7% for PVC and idiopathic VT, 77.4% for SHD related VT	29.6% for PVC and idiopathic VT, 32% for SHD related VT	5%	Yes, in 10% for SHD related VT	Yes, 14% for PVC and idiopathic VT, 10% for SHD related VT	8.3 ± 4 min and 25.4 ± 14 Gycm^2^ when used for PVC and idiopathic VT; 21.2 ± 5 min and 48.6 ± 32 Gycm^2^ when used in SHD related VT	Yes, 100%
Dinov et al., 2014	F	227	Prospective	SHD related VT (NICM, ICM)	181 ± 63.6 min for NICM and 155 ± 49 min for ICM	66.7% for NICM and 77.4% ICM	59.5% for NICM and 43% for ICM	11.1% for NICM and ICM	Yes, 9%	Yes, 100% of procedures	39 ± 22.4 min for NICM and 26 ± 19.1 min for ICM, dose not reported	No
Tanawuttivat et al., 2015	F	815 in 8 different studies	Review with analysis	Idiopathic/outflow tract VT	Not reported in the review	76–100%	0–23%	0–3%	No	Yes	Not reported in the review	No
Kumar et al., 2016	F	695	Retrospective, consecutive pts	Idiopathic VT and SHD related VT (NICM, ICM)	Not reported	79% for idiopathic VT, 60% for NICM and 56% for ICM	At 6 years FU: 23% for idiopathic VT, 62% for NICM and 46% for ICM	3.7% for idiopathic VT, 6.7% for NICM, 8.3% for ICM	Yes, 3% for idiopathic VT, 30% for NICM and 8% for ICM	Yes	31.3 ± 20 min for idiopathic VT, 43 ± 21.6 for NICM and 45.1 ± 30.2 for ICM, dose not reported	No
Lamberti et al., 2015	ZF	19	Retrospective, consecutive pts	PVC, idiopathic VT	170.2 ± 45.7 min	100%	10.5%	0	No	No	0	Yes, 100%
Sanchez et al., 2016	ZF	16	Retrospective, consecutive pts	PVC, Idiopathic VT and SHD related VT (aetiology not specified)	150 ± 45 min	100%	Not reported	0	No	No	0	Yes, 75%
Razminia et al., 2017	ZF	44	Retrospective, consecutive pts	PVC, Idiopathic VT and SHD related VT (ICM)	201.9 min for PVC, 257.5 min for idiopathic VT and SHD related VT	93.3% for PVC, 100% for idiopathic VT and SHD related VT	6.6 for PVC, 21.4 for idiopathic VT and SHD related VT	3.3% for PVC, 0 for idiopathic VT and SHD related VT	No	No	0	Yes, % not specified
Giaccardi et al., 2019	ZF	7	Retrospective	PVC, VT (aetiology not specified)	75 min for PVC, 128 ± 21 min for VT	100%	0	0	No	No	0	No
Sadek et al., 2019	ZF	10	Prospective	PVC, SHD related VT (aetiology not specified)	Not reported	100%	0 for PVC, 17% for SHD related VT	0	No	No	0	Yes, 100%
Cano et al., 2016	MinF	41	Prospective	SHD related VT (NICM, ICM)	204 ± 61 min	66%	7.3%	4.9%	Yes, 16%	Yes	6.08 (1.51–12.36) min and 10.8 (2.9–41.1) Gycm^2^	No
Wang et al., 2017	MinF	160	Prospective, multi-centre	PVC, idiopathic VT	77.1 ± 33.8 min	84.1%	1.9%	1.2%	No	Yes, 5.6% of procedures	1.5 ± 0.3 min when used, dose not reported	No

*ZF* zero fluoroscopy, *MinF* minimal fluoroscopy, *F* fluoroscopy, *VA* ventricular arrhythmia, *OSR* overall procedural success rate, *RR* recurrence rate, *CR* complication rate, *ICE* intracardiac echocardiography, *PVC* premature ventricular contractions, *VT* ventricular tachycardia, *SHD* structural heart disease, *NICM* non-ischaemic cardiomyopathy, *ICM* ischaemic cardiomyopathy, *FU* follow-up, *min* minutes, *Gycm*^*2*^ Gray centimeter squared

### Clinical implications

Our study shows that completely ZF approach to CA is feasible and safe in most types of VAs even when SHD is present. Furthermore, procedural outcomes of ZF approach are not inferior to the CF-based procedures. However, to achieve these comparable results reliance on the available ZF oriented technology, namely ICE and available 3D EAM systems, is mandatory. We routinely used ICE in all ablation procedures which possibly contributed to our low CR and relatively high OSR with short TPT. Utilisation of ICE seems to eliminate the need for X-ray fluoroscopy by allowing real-time imaging of catheters during mapping and ablation, monitoring of possible procedural complications (especially pericardial effusion) and direct imaging of anatomical variations and structures (i.e. papillary muscles) that cannot be adequately imaged with the 3D EAM systems [32].

Our results appreciate the inherent limitations of the ZF approach in treatment of VAs. With primarily using 3D EAM system and ICE, there seem to be two major limitations, where the use of fluoroscopy cannot be avoided:

1. Epicardial mapping and ablation and the need to visualise wires, long sheaths and catheters in the epicardial space.
2. Ablation of VAs originating in the LV summit where visualisation of coronary anatomy in necessary to avoid RF delivery close or on top of a large caliber coronary artery.

### Study limitations

There are some study limitations decreasing the value of our conclusions. Firstly, this study is observational without a control group and it is not randomised to compare the CF and ZF approaches. Secondly, all procedures were performed by a single operator, already experienced in fluoroless CA of different tachycardias. On the other hand, the fact that a single operator has done all the procedures can be considered a partial advantage in avoiding inter-operator variability. Thirdly, the follow-up period for the detection of possible recurrences of arrhythmias was relatively short, albeit comparable to the available published data. It is reasonable to assume that with a longer follow-up more patients would have experienced arrhythmia recurrences. Fourthly, relatively small study population precluded detailed analysis of VA subgroups, which limits the value of our analysis. Finally, several patient-related factors, namely metabolic and inflammatory status, could affect the electrophysiological properties of the myocardium and outcomes of ablation procedures [33, 34]. However, a relatively small study population precluded detailed analysis of the potential impact of these factors on procedural outcomes in different VA subgroups.

## Conclusions

Fluoroscopy minimising approach for CA of VAs is feasible and safe in patients with SHD and SNH. Although ZF approach relying only on 3D EAM system and ICE can be utilised in most cases, fluoroscopy could not be completely abolished in VAs with epicardial and LV summit substrate location.

## Data Availability

Data generated or analysed during this study is for the most part included in this published article. Additional raw data is available and can be shared upon request to the corresponding author.

## Abbreviations

CA: Catheter ablation
CF: Conventional fluoroscopy
VA: Ventricular arrhythmia
AF: Atrial fibrillation
ALARA: As low as reasonably achievable
NZF: Near-zero fluoroscopy
ZF: Zero fluoroscopy
3D: Three-dimensional
EAM: Electro-anatomical mapping
SVT: Supra-ventricular arrhythmia
SNH: Structurally normal heart
SHD: Structural heart disease
ECG: Electrocardiogram
AAD: Anti-arrhythmic drug
CIED: Cardiac implantable electronic device
CT: Computed tomography
MRI: Magnetic resonance imaging
USA: United States of America
ICE: Intra-cardiac echocardiography
RF: Radio-frequency
LAVA: Local abnormal ventricular activity
LP: Late potential
RV: Right ventricle
VT: Ventricular tachycardia
W: Watt
LV: Left ventricle
DAP: Dose area product
TSP: Trans-septal puncture
TPT: Total procedural time
OSR: Overall procedural success rate
RR: Recurrence rate
CR: Complication rate
ATP: Anti-tachycardia pacing
NY: New York
SD: Standard deviation
ICM: Ischaemic cardiomyopathy
NICM: Non-ischaemic cardiomyopathy
IHD: Ischaemic heart disease
RVOT: Right outflow tract
LCX: Left circumflex coronary artery
RAO: Right anterior oblique
RA: Right atrium
CS: Coronary sinus
GCV: Great cardiac vein
SVC: Superior caval vein
IVC: Inferior caval vein
LVEF: Left ventricular ejection fraction
LVEDV: Left ventricular end-diastolic volume
